# Analgesic efficacy of ultrasound guided erector spinae plane block versus serratus anterior plane block in pediatric patients undergoing aortic coarctectomy; a randomized controlled study

**DOI:** 10.1186/s12871-025-03256-y

**Published:** 2025-07-30

**Authors:** Ahmed Ali Mohamed Gado, Mahmoud Abdeltawab Mahmoud Atia, Ahmad Awad Roman, Wafaa Mohamed Elsadeq, Victor Farouk Jaccoub

**Affiliations:** Anesthesiology, Surgical Intensive Care and Pain Management Department, Faculty of Medicine, Cairo University, Cairo, Egypt

**Keywords:** Erector spinae block, Serratus anterior block, Pediatric, Aortic coarctectomy

## Abstract

**Background:**

Aortic coarctectomy (AC) is associated with marked intraoperative hemodynamic alterations and significant post-thoracotomy pain. In this study, the analgesic effects of erector spinae plane block (ESPB) and serratus anterior plane block (SAPB) were compared.

**Methods:**

28 pediatric patients were randomized into the ESPB group (received unilateral ESPB using a volume of 0.4 ml/kg of bupivacaine 0.25% and lidocaine 1% on the same side of the planned thoracotomy) and the SAPB group (received unilateral block by injecting the same local anesthetic volume and mixture at the level of the 5th rib). Both blocks were given after anesthetic induction. The primary endpoint was total intraoperative fentanyl dose, while postoperative pain scores and the first 24 h morphine dose were secondary endpoints.

**Results:**

Intraoperative fentanyl consumption (mcg/kg) didn’t show a significant difference between the ESPB group (1.21 ± 0.43) and the SAPB group (1.36 ± 0.5), mean difference = 0.14, 95% CI (-0.21 to 0.50), p value = 0.421. Pain scores and first 24 h. morphine dose after surgery were comparable between both groups, *p* > 0.05.

**Conclusion:**

Both SAPB and ESPB provided comparable perioperative analgesia in pediatric AC, which was reflected by comparable intraoperative fentanyl dose, postoperative pain scores, and postoperative morphine dosage.

**Trial registration:**

NCT06567275, trial registration date: (22-08-2024)

**Supplementary Information:**

The online version contains supplementary material available at 10.1186/s12871-025-03256-y.

## Introduction

Aortic coarctectomy (AC) is a corrective pediatric cardiac surgical procedure done through a left lateral or postero-lateral thoracotomy incision. It is associated with marked intraoperative hemodynamic alterations related to high intraoperative nociception and aortic clamping and declamping [1].

Also, the significant pain after thoracotomy may elevate the risk of postsurgical pulmonary problems, postoperative mortality, prolonged hospital stay, and increased usage of healthcare supplies [2, 3].

Management of perioperative pain in such a situation could be achieved by high-dose opioids, which are a strategy that gained popularity in the last years [4] due to their beneficial effects, including hemodynamic stability and stress response reduction. However, excess sedation, respiratory depression, and extended mechanical ventilation are major concerns [5].

In pediatric cardiac surgery, regional analgesia has been largely accepted as an essential component of multimodal analgesia because it is known to reduce the neuro-endocrine stress response, offer superior analgesia, and accelerate extubation after surgery [6].

Erector spinae plane block (ESPB) was thought to be an appropriate technique for postoperative pain management, offering efficient analgesia with little need for extra adjuncts in pediatric patients undergoing thoracotomy or sternotomy [7–10].

Serratus anterior plane block (SAPB) is a promising fascial plane block with.

technical simplicity and relative safety, which relieves pain of the ipsilateral hemithorax [11, 12]. A recent meta-analysis revealed that SAPB offered a decrease in narcotic dosage and pain score within the first day after pediatric.

cardiothoracic thoracotomy surgery [13].

To our knowledge, the comparison of SAPB versus ESPB in AC operations in pediatric patients has not been investigated yet. In this study, the analgesic effects of ESPB and SAPB in pediatric patients undergoing AC through thoracotomy were compared. The primary endpoint was the total intraoperative fentanyl consumption, with secondary endpoints being hemodynamics, time to first opioid request, postoperative opioid dose, and adverse effects.

### Patients and methods

This prospective randomized double-blinded study had been approved by our tertiary university ethics and research committee (ID: MD-316-2023) and was registered on the Clinical Trials Registry (ID: NCT06567275) before enrollment of the patients. The study adhered to the Consolidated Standards of Reporting Trials for randomized trials (CONSORT) guidelines. Prior to surgery, each patient’s parents or guardians provided written informed consent. The study included 28 pediatric patients aged between 3 months and 2 years, with a Risk Adjustment for Congenital Heart Surgery (RACHS-1) score of 3, and scheduled for AC through a thoracotomy incision in the pediatric cardiothoracic operation theater (Pediatric Specialized Hospital, Cairo University) during the period from August 2024 to April 2025.

### Exclusion criteria

Patients whose parents declined to participate, mechanical ventilation or inotropic infusion before surgery and those with perioperative cardiopulmonary arrest. Patients undergoing AC with sternotomy incision or with a history of mental retardation or developmental delay affecting pain assessment. Coagulopathy (PT < 75% of control), congenital anomalies or infection at the injection site, allergies to the studied drugs, elevated liver enzymes, renal impairment (Creatinine value more than 1.2 mg/dl or BUN more than 20 mg/dl), and heart failure or redo patients and previous catheter dilatations were also excluded.

### Randomization-Blinding

Randomization was done through a computer-generated sequence (through the randomizer.org online website), which was kept in sealed opaque envelopes, ensuring equal allocation of participants to either the SAPB group or the ESPB group. To achieve double blinding, parents didn’t know the type of the block that was given during the operation, and both blocks were given by an experienced consultant who had no further role in this study and performed the blocks in alignment with the recognized standards. The anesthetic management was standardized and performed by anesthesiologists who were blinded to group assignment as they left the room during the block performance. Also, the intraoperative data collectors and the postoperative outcome assessors (ICU staff and analgesia administrators) were blinded to group allocation.

### Preoperative assessment

After all patients and their parents arrived in the pre-anesthesia room one hour prior to the procedure and gave their informed consent, a thorough history from the parents was taken, followed by a thorough clinical examination of the child, and then investigations were checked. 0.08 mg/kg midazolam and 0.01 mg/kg atropine were given intramuscularly 20 min prior to anesthetic induction as a premedication.

### Intraoperative

After applying non-invasive blood pressure, pulse oximetry, and ECG, anaesthetic induction was done by sevoflurane 5% in 50% O₂ followed by IV cannula insertion and fentanyl (1 mcg/kg) IV administration. IV atracurium 0.5 mg/kg was administered to facilitate intubation (a capnogram was connected to monitor End-tidal CO₂) and atracurium infusion in a dose of 0.5 mg/kg/h was used to maintain muscle relaxation together with sevoflurane 2% for maintenance of anesthesia.

Mechanical ventilation was achieved by using a pressure-controlled mode in which the pressure was adjusted to establish a tidal volume of 7–10 ml/kg and the rate was adjusted to keep end-tidal CO₂ between 30 and 40 mmHg.

A temperature probe (nasopharyngeal) was placed; a central venous catheter and a right upper limb arterial cannula with or without a femoral arterial cannula were introduced, and they were connected to the invasive blood pressure dome.

### Study groups

The patient’s position was changed to the right lateral position, and cases were allocated to one of the following groups:

1. ESPB group: 14 patients received ultrasound (US)-guided unilateral ESPB using a volume of 0.4 ml/kg of bupivacaine 0.25% and lidocaine 1% on the same side of the planned thoracotomy as follows: Aseptic precautions were followed; the T5 spinous process (SP) was determined by palpation down from the C7 SP. A high-frequency 12 MHz linear US probe (S-Nerve Ultrasound System P07576, USA with SL Ax/6-13 MHz linear high-frequency transducer) was placed in a longitudinal orientation 3 cm lateral to the T5 SP correlating with the T4 transverse process (TP). The trapezius (uppermost), rhomboids major (middle), and erector spinae (lowermost) muscles were visualized superior to the hyper-echoic TP. Through an in-plane technique, a needle (25-gauge, 2.5–5 cm short beveled needle or blunt-tipped hypodermic needle) was introduced in the caudo-cephalad orientation till its tip reached deep to the erector spinae muscle, confirming this by injecting 0.5 ml saline before administration of local anesthetic mixture [8].
2. SAPB group: 14 patients received US-guided SAPB using a volume of 0.4 ml/kg of bupivacaine 0.25% and lidocaine 1% on the same side of the planned thoracotomy as follows: Aseptic precautions were followed; a high-frequency 12 MHz linear US probe (S-Nerve Ultrasound System P07576, USA with SL Ax/6-13 MHz linear high-frequency transducer) was placed in a sagittal plane over the mid-clavicular part of the thorax cage. Ribs were counted infero-laterally till the 5th rib in the mid-axillary line. The latissimus dorsi (superficial and posterior), teres major (superior), and serratus anterior muscle (SAM) (deep and inferior) were visualized overlying the 5th rib. A needle (22-gauge, 2.5–5 cm short beveled needle or blunt-tipped hypodermic needle) was inserted in a perpendicular way through the skin to contact the rib, then it was withdrawn 1–2 mm to be between the SAM and the rib, confirming this by injecting 0.5 ml saline before administration of the local anesthetic mixture [14].

In both groups, the skin incision was allowed to start 20 min after the block, and the end-tidal sevoflurane concentration was maintained between 0.7 and 1.3 of minimum alveolar concentration (MAC), guided by a hemodynamic target within 20% of baseline value. Increased HR and/or SBP above 20% of baseline values recorded 5 min after intubation was managed first by increasing the sevoflurane concentration to obtain an end-tidal value of at least 1.3 MAC; if no response occurred, boluses of 1 mcg/kg fentanyl were given after excluding surgical issues.

Cases that would need > 2 mcg/kg fentanyl dose during skin incision or rib retraction would be excluded due to a possibility of unsuccessful block.

IV 1–2 mg/kg heparin was administered 10 min before aortic cross-clamping to achieve an activated clotting time > 200 s.

Elevated blood pressure after aortic cross-clamping was initially managed by one bolus of fentanyl to be followed by 0.5 mcg/kg/min infusion of sodium nitroprusside, increased according to the response in unresponsive cases.

Sodium nitroprusside infusion was stopped one minute before aortic declamping. Post-declamping hypotension was managed by 1–2 ml of calcium gluconate and 10 ml/kg of isotonic saline. If hypotension persisted, a bolus of 5–10 mcg intravenous norepinephrine was given. If the patient was still hypotensive, an infusion of 0.25 mcg/kg/min of norepinephrine was started untill stabilization of blood pressure.

Finally, inhalational anesthesia was discontinued at the surgery’s end. And muscular blockade was reversed by atropine 0.01 mg/kg and neostigmine 0.05 mg/kg. Extubation was performed on those who were fully awake, expressing eye-opening and purposeful movement with an adequate breathing pattern.

### Postoperative

All patients were discharged to the pediatric ICU and managed by a standardized protocol. IV paracetamol 15 mg/kg/6 h was given. Postoperative pain was assessed using the Face, Legs, Activity, Cry, Consolability (FLACC) score every 2 h for the first 24 h postoperatively. If the FLACC score ≥ 4, 0.02 mg/kg morphine IV was administered as rescue analgesia to be repeated every 15–20 min untill the pain score reaches < 4, not exceeding 0.2 mg/kg every 6 h [15].

### Measurements

#### Intraoperative

The total intraoperative fentanyl consumption was recorded, and vital signs, including HR and SBP from the radial arterial line, were recorded at (T_1;_ baseline reading 5 min after intubation, T_2;_ before skin incision 15 min after the block, T_3_; after skin incision, T_4;_ after rib retraction, T_5;_ after aortic clamping, T_6;_ after aortic declamping, T_7;_ immediately after skin closure and T_8;_ 15 min after extubation). The need for and the dose of direct vasodilator (sodium nitroprusside) after aortic clamping were also recorded.

#### In the pediatric ICU

Pain scores, the time to 1 st morphine rescue analgesia (time between block administration and a patient FLACC score > 4), and the first 24 h. morphine dose. Vital signs, including HR and SBP, were recorded every 2 h for the first 6 h postoperatively. The time spent in the ICU was recorded.

#### Outcomes

The primary endpoint was total intraoperative fentanyl dose, while all other intraoperative and postoperative measurements were secondary outcomes.

#### Sample size

We conducted a pilot study (8 cases per group) in which the reported mean ± SD intraoperative fentanyl consumption (1^ry^ outcome) was 3 ± 0.5 mcg/kg in the ESPB group and 2.25 ± 0.7 mcg/kg in the SAPB group. Using the G power program, a two- tailed t-test; a minimum number of 24 patients (12 patients per group) was calculated to have a study power of 80% and an alpha error of 0.05. The number was increased to 28 patients (14 patients per group) to compensate for possible dropouts.

#### Statistical analysis

Statistical Package for Social Science (SPSS) software, version 15 for Microsoft Windows (SPSS Inc., Chicago, IL, USA), was used for data analysis. Categorical data was presented as frequency (%) and analyzed by chi-square test. Continuous data was checked for normality using the Shapiro-Wilk test. Normally distributed data were presented as means ± standard deviations and analyzed using an unpaired t-test. Skewed data were expressed as medians (quartiles) and analyzed using the Mann-Whitney test. Repeated measures were analyzed using analysis of variance (ANOVA) for repeated measures with post-hoc using the Bonferroni test to adjust for multiple comparisons. Survival analysis (Kaplan-Meier curve) was used to analyze the time to first opioid request in both groups. A P-value less than 0.05 was considered statistically significant.

## Results

**Fig. 1:**
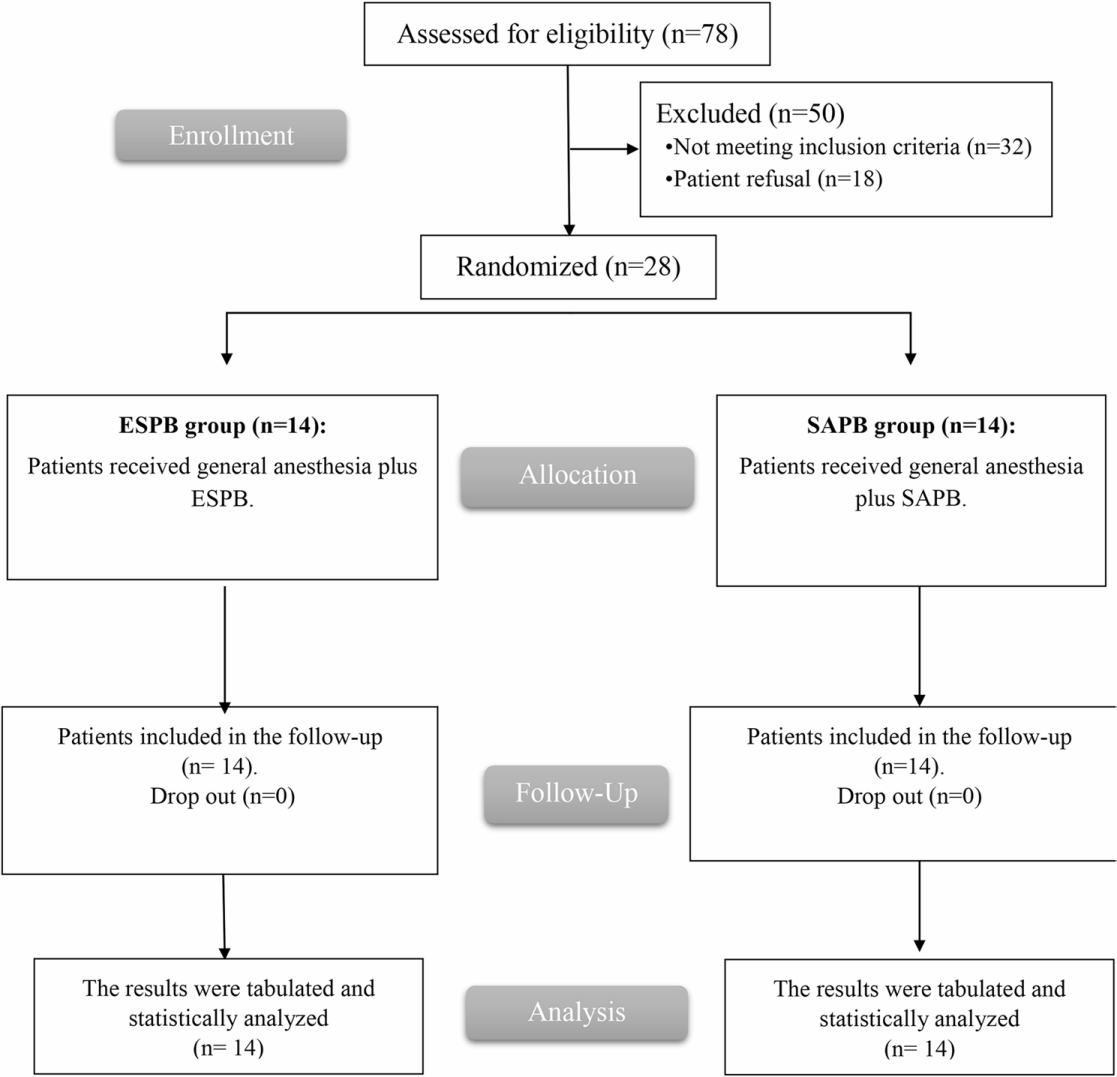
CONSORT flowchart of both groups

The eligibility of 78 pediatric patients was assessed; 32 patients didn’t fulfill the inclusion criteria, and 18 patients refused to share in this study. The remaining 28 patients were randomly and equally divided into the ESPB group and the SAPB group. All patients were followed up, and their data were analyzed without dropouts. Figure 1.

Demographic data and ICU stay duration were comparable between both groups. **(**Table 1**)**

**Table 1:** Demographic data and ICU stay time

	Group ESPB (*n* = 14)	Group SAPB (*n* = 14)	*P* value
Gender Male Female	8 (57.1%) 6 (42.9%)	7 (50%) 7 (50%)	0.985
Age in months	4.6 ± 1.7	4.8 ± 1.7	0.712
Weight in kg	4.6 ± 0.99	4.7 ± 1.1	0.719
ICU stay time in hours	59.4 ± 22.5	58.3 ± 19.8	0.888

Data are presented as mean ± SD or frequency (%)

*ESPB*, erector spinae plane block, *SAPB*, serratus anterior plane block

### The primary outcome: intraoperative consumption of fentanyl

Intraoperative fentanyl consumption (mcg/kg) didn’t show a significant difference between the ESPB group (1.21 ± 0.43) and the SAPB group (1.36 ± 0.5), mean difference = 0.14, 95% CI (−0.21 to 0.50), p value = 0.421. **(**Table 2**)**

**Table 2:** Intraoperative data and postoperative morphine consumption

	Group ESPB (*n* = 14)	Group SAPB (*n* = 14)	*P* value
Intraoperative fentanyl	1.21 ± 0.43	1.36 ± 0.5	0.421
Need for sodium nitroprusside Yes No	2 (14.3) 12 (85.7)	9 (64.3) 5 (35.7)	0.007*
If yes, amount in microgram/kg/min	1.25 ± 0.41	1.57 ± 0.54	0.089
Amount of morphine for 1 st 24 h	0.273 ± 0.05	0.269 ± 0.06	0.883

Data are presented as mean ± SD or frequency (%),

*ESPB* erector spinae plane block, *SAPB* serratus anterior plane block

* Significant p value less than 0.05

### Perioperative vital signs

**Fig. 2:**
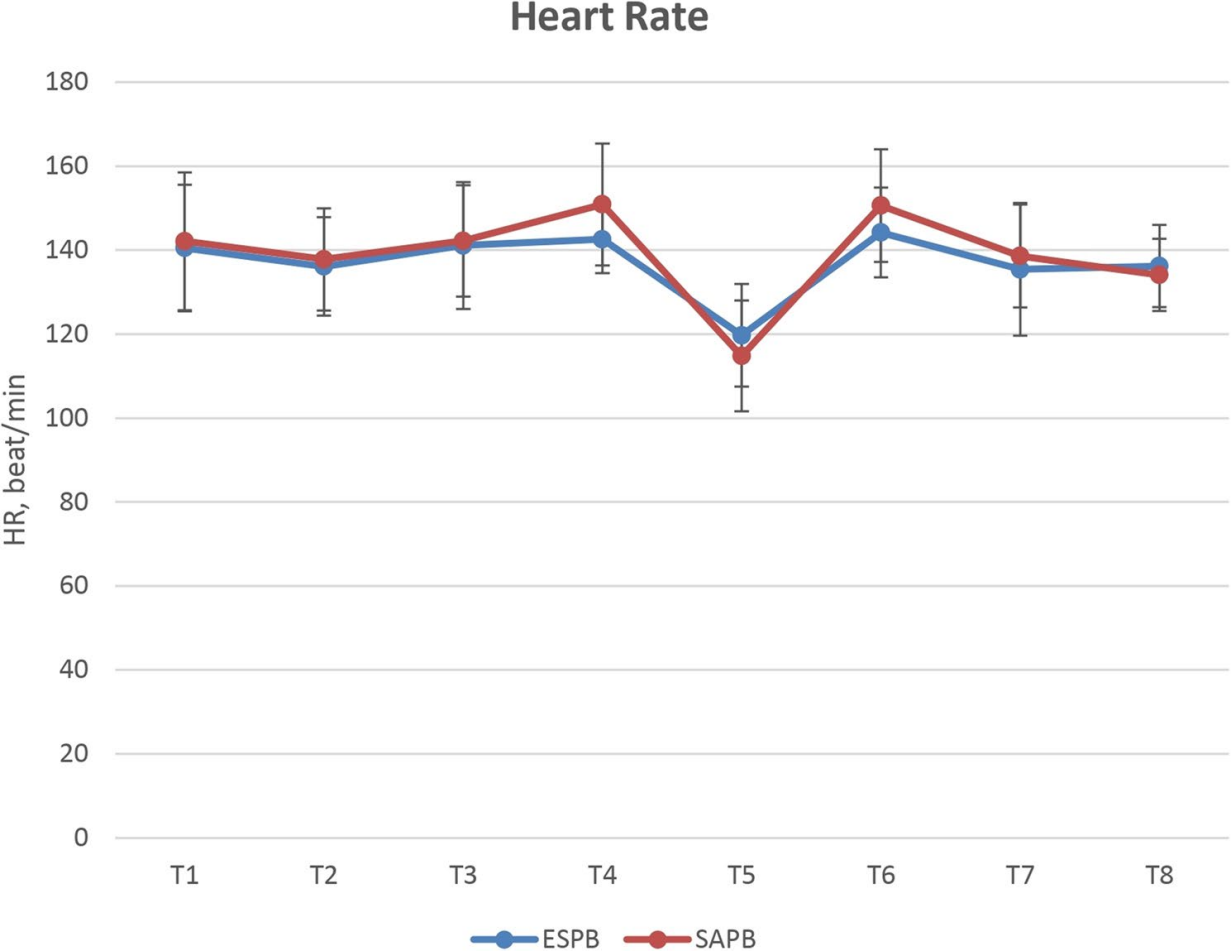
Intraoperative heart rate in both groups (T_1;_ baseline reading 5 min after intubation, T_2;_ before skin incision 15 min after the block, T_3_; after skin incision, T_4;_ after rib retraction, T_5;_ after aortic clamping, T_6;_ after aortic declamping, T_7;_ immediately after skin closure and T_8;_ 15 min after extubation). The points represent the mean values, and the vertical bars represent the standard deviation

**Fig. 3:**
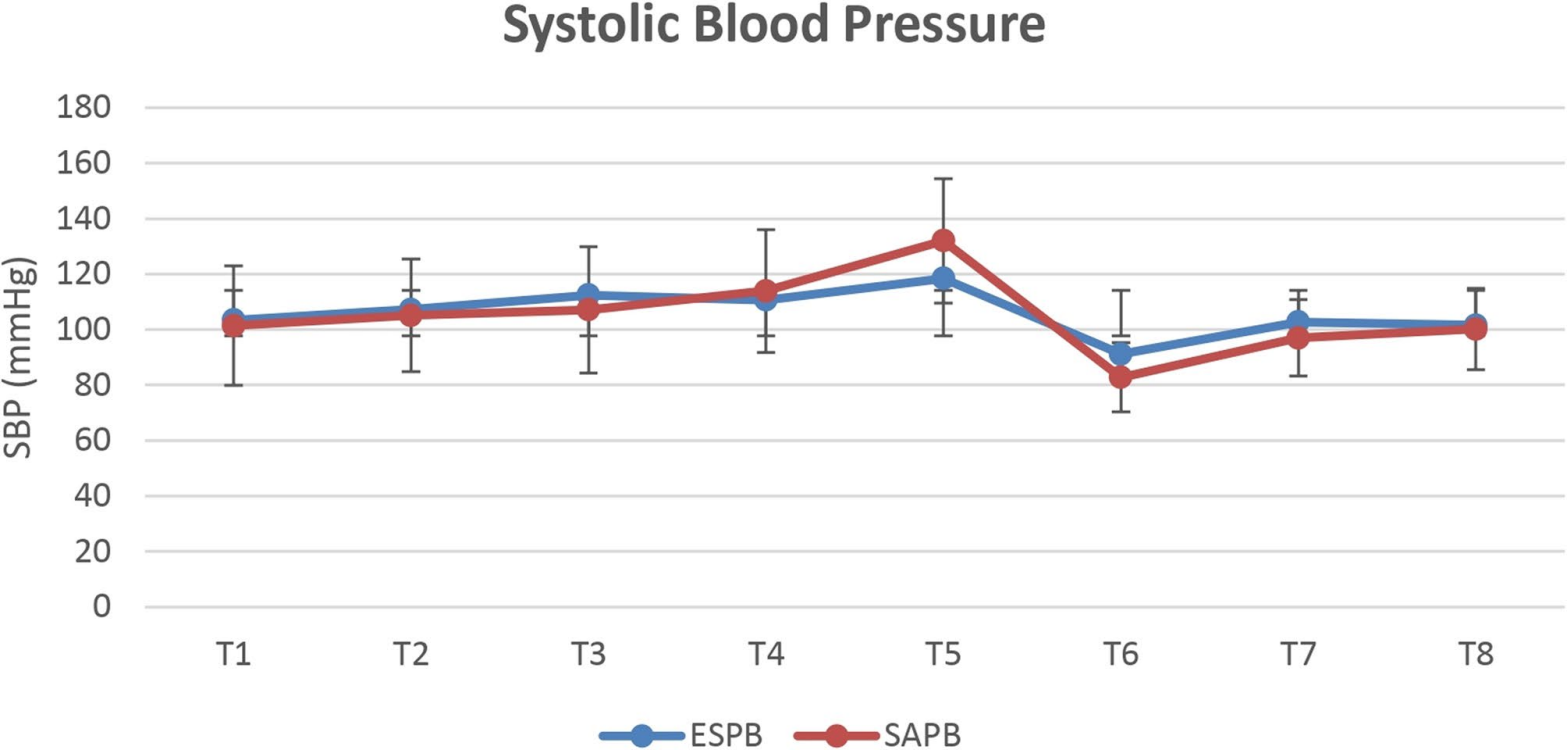
Intraoperative systolic blood pressure in both groups; (T_1;_ baseline reading 5 min after intubation, T_2;_ before skin incision 15 min after the block, T_3_; after skin incision, T_4;_ after rib retraction, T_5;_ after aortic clamping, T_6;_ after aortic declamping, T_7;_ immediately after skin closure and T_8;_ 15 min after extubation). The points represent the mean values, and the vertical bars represent the standard deviation

Intraoperative HR and SBP values were comparable between both groups. **(**Figs. 2 and 3**)** A significantly higher number of patients in the SAPB group required sodium nitroprusside than in the ESPB group (64.3% vs. 14.3%, *p* = 0.007); however, the mean ± SD infusion dose of sodium nitroprusside was comparable between both groups *p* = 0.089. **(**Table 2**)** Also, the postoperative HR and SBP values were comparable between both groups.

### Postoperative analgesic outcomes

**Fig. 4:**
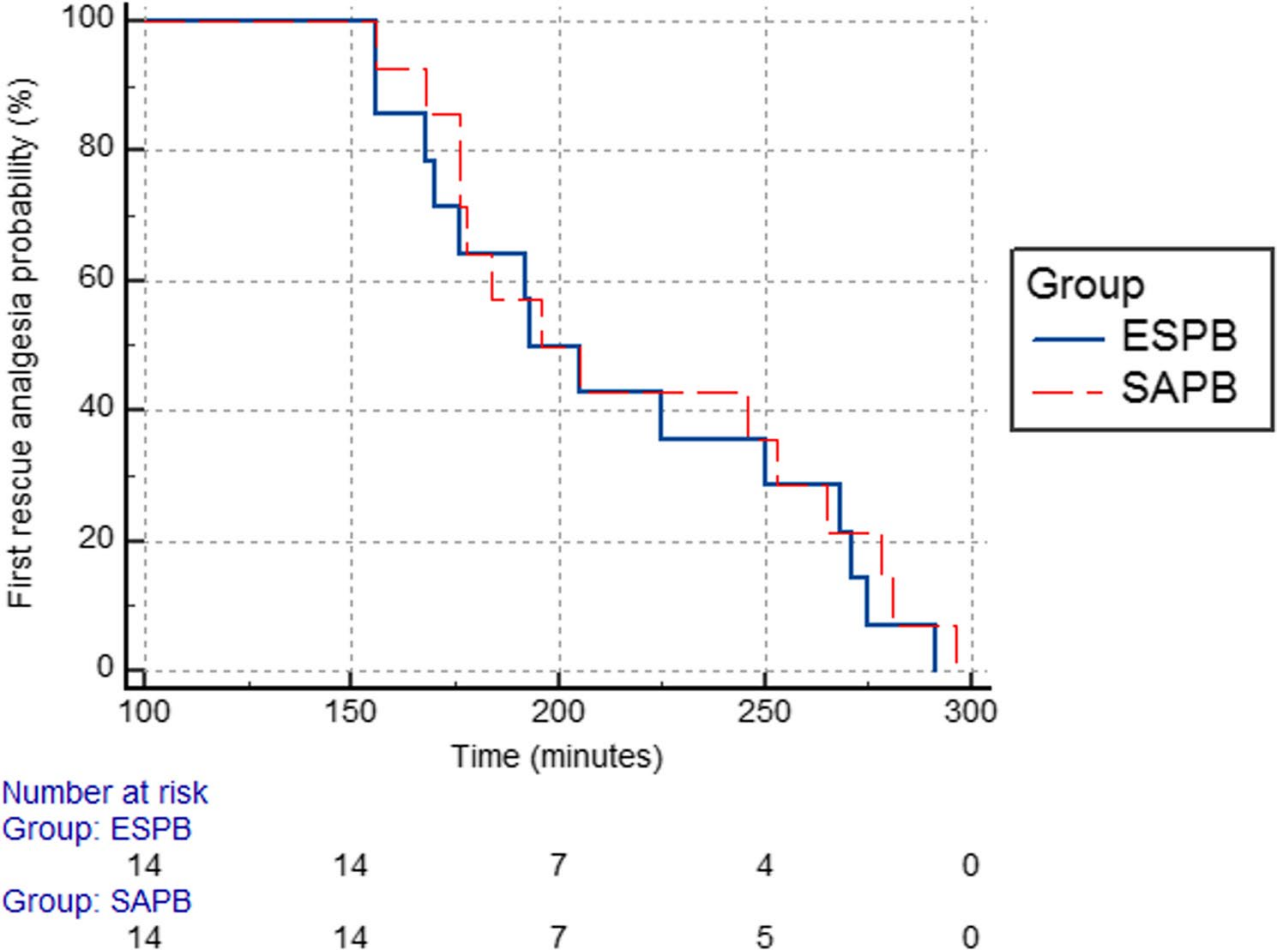
Kaplan Meier curve for the time (in minutes) to first rescue analgesia

Regarding FLACC pain scores, there were no significant differences between both groups at any time point of assessment. **(**Table 3**)** The amount of morphine consumption in the first 24 h. after surgery was comparable between both groups, *p* = 0.883. Also, survival curve analysis showed that the first rescue analgesic time was insignificantly different between the ESPB group (214 ± 48.4) minutes and the SAPB group (203.57 ± 44.84) minutes, *p* = 0.495. **(**Fig. 4**)** No block-related hematoma or local anesthetic toxicity was reported in any case.

**Table 3:** Postoperative FLACC pain scores

	Group ESPB (*n* = 14) Mean ± SD	Group SAPB (*n* = 14) Mean ± SD	*P* value*
FLACC scores after 2 h.	2.7 ± 1.06	2.5 ± 0.5	0.506
FLACC scores after 4 h.	3.2 ± 1.4	3.8 ± 1.4	0.249
FLACC scores after 6 h.	4.5 ± 1.4	4.2 ± 1.7	0.639
FLACC scores after 8 h.	2.8 ± 1.02	2.3 ± 0.5	0.113
FLACC scores after 10 h.	3.1 ± 1.1	2.6 ± 0.5	0.090
FLACC scores after 12 h.	2.9 ± 1.2	2.8 ± 0.8	0.715
FLACC scores after 14 h.	3.2 ± 1.05	3.8 ± 1.2	0.149
FLACC scores after 16 h.	3.1 ± 1.5	2.9 ± 1.3	0.704
FLACC scores after 18 h.	3.1 ± 1.1	3.07 ± 1.1	0.867
FLACC scores after 20 h.	3.2 ± 1.5	2.5 ± 0.8	0.139
FLACC scores after 22 h.	3.6 ± 1.5	3.5 ± 1.4	1.00
FLACC scores after 24 h.	3.2 ± 1.1	3.8 ± 1.7	0.249

Data are presented as mean ± SD

FLACC: Face, Legs, Activity, Cry, Consolability scale, ESPB: erector spinae plane block, SAPB: serratus anterior plane block

## Discussion

The findings of this study revealed that SAPB provided comparable perioperative analgesia to ESPB in pediatrics undergoing AC via thoracotomy. This was achieved by the comparable intraoperative fentanyl consumption, postoperative pain scores, 24 h opioid consumption, and perioperative hemodynamics, with a smaller number of patients in the ESPB group requiring sodium nitroprusside than in the SAPB group.

Adequate perioperative analgesia in pediatric patients undergoing thoracotomy is necessary to decrease the perioperative neuro-hormonal response and cardiopulmonary complications [16].

The multifactorial thoracotomy pain from the surgical wound, pleural and muscular trauma, rib disruption, and intercostal nerve injury, and the presence of thoracotomy tubes require a multimodal approach to effectively handle the intraoperative nociception and manage postoperative pain [2].

Incorporation of regional analgesic techniques in such an approach ensures optimal analgesia in pediatric cardiac surgeries, reduces opioid consumption, and enhances postoperative recovery [9]. In the context of coarctation repair, this is crucial to prevent hemodynamic instability and improve patient outcomes.

Although many studies have been published analyzing the effectiveness of each form of block as a beneficial analgesic strategy, no previous studies compared both types of blocks in AC.

Regarding the use of SAPB in pediatric thoracic surgery, a previous study revealed that it provided a safe, efficient, and simple regional approach for thoracotomies, as it reduced the intraoperative and postoperative opioid dose and pain scores after surgery [17].

Other analgesic findings were reported by Chen C et al. [18] who evaluated the role of SAPB in pediatric ear reconstruction via costal cartilage harvest and demonstrated superior analgesia compared to incision site local anesthetic infiltration.

Furthermore, Kaushal B et al. [19] compared SAPB, pectoral nerves II block (PEC), and intercostal nerve block for postoperative thoracotomy analgesia in pediatric cardiac surgery, and their results revealed a comparable analgesic profile between SAPB and PEC block with superior analgesia to intercostal nerve block.

SAPB acts by blocking the lateral cutaneous branches of the 2nd to 9th spinal intercostal nerves (T2 to T9), resulting in paresthesia of the anterolateral thorax [14].

Forero et al. initially reported using ESPB in 2016 to treat chronic thoracic neuropathic pain [20]. Its function in perioperative pain management during cardiac procedures involving median sternotomy was first presented by Nagaraja et al. in 2018 [21].

Regarding ESPB’s role in pediatric thoracic surgeries, a previous case report done by Holladay JD et al. revealed that continuous ESPB adequately controlled pain and decreased opioid usage in an 18-year-old case of complex AC via lateral thoracotomy [22]. Another case report for continuous ESPB in a lung-transplanted, anticoagulated pediatric patient revealed an adequate analgesic quality [23]. Also, ESPB provided comparable analgesia to the epidural block after thoracotomy with a lower complication rate [10].

The ESPB acts by blocking the thoracic intercostal nerves extending 3–4 levels cranially and caudally from the injection site. Local anesthetics spreading through channels in the inter-transverse area to the paravertebral space may extend their effect to act on dorsal and ventral rami of the spinal nerve roots [24]. The spread may also occur via the costo-transverse foramen [25] or extend to the epidural space [26], resulting in some degree of sympathetic blockade, which reflects the significantly lower number of cases that required vasodilators after cross-clamping in the ESPB group. Aortic cross-clamping is known to produce detrimental hemodynamic effects, as it typically produces an increase in the proximal blood pressure, increases myocardial afterload, and may increase the risk of stroke. Furthermore, baroreceptor adaptation in patients with AC may be associated with elevated levels of norepinephrine [1]. In our study, ESPB was done at T5, which could cover a dermatome area from T1 up to T9. So, we suppose that this sympathetic modulation via ESPB (through epidural or paravertebral spread) may mitigate the hemodynamic effects of aortic cross-clamping by producing vasodilatation in the collateral circulation of the great vessels (T1 to T4 sympathetic innervation) with or without some degree of vasodilatation in the mesenteric circulation (T5 to T9 sympathetic innervation).

Previous studies comparing both types of blocks showed inconsistent results; Wang HJ et al. [27] compared these two block types in patients undergoing radical mastectomy, and their results revealed comparable analgesic and safety profile for both blocks. Also, Wu W et al. [28] revealed that both the ESPB and SAPB demonstrate effective reduction in postoperative opioid consumption and the need for rescue analgesics compared to the control group for postoperative analgesia in uniportal thoracoscopic lobectomy.

However, previous reports revealed a superior analgesic profile for ESPB as compared to SAPB in adult patients scheduled for thoracoscopic surgery [29], posterolateral thoracotomy [30], and minimally invasive thoracic surgery [31] or video-assisted thoracoscopy [32]. Different age and surgical type may explain the difference from our results.

Limitations.

There were some limitations for this study; first, the plasma levels of local anesthetic were not measured to evaluate the risk of anesthetic toxicity. Second, obviously a single shot of regional block has a shorter duration of action compared to continuous infusion. Third, the small sample size may limit the generalizability of our findings. Also, the pilot study suggested a larger difference in fentanyl use between both groups than what was observed. So, it may have been underpowered to detect smaller, yet potentially clinically meaningful, differences in fentanyl use. Fourth, pain evaluation in pediatrics in this age group is a challenge and has inherent limitations. Observational scales like FLACC depend on behaviors, and they may not be able to distinguish between pain-related and non-pain-related behaviors, with the potential for misinterpretation. Fifth: Lack of control group, but for ethical and practical considerations, we aimed to provide adequate intraoperative analgesia to minimize adverse effects and allow rapid recovery for all cases. Last, we couldn’t manage the intraoperative anesthetic depth with BIS monitoring, and we depended on the end-tidal sevoflurane concentration for fentanyl administration.

## Conclusion

Both SAPB and ESPB provided comparable perioperative analgesia in pediatric AC, which was reflected by comparable intraoperative fentanyl consumption, postoperative pain scores, and postoperative morphine dosage.

## Supplementary Information

Supplementary Material 1.

Supplementary Material 2.

Supplementary Material 3.

Supplementary Material 4.

## Data Availability

•The datasets used and/or analysed during the current study are available from the corresponding author on reasonable request.
