# Supplementary material for: Analgesic efficacy of ultrasound guided erector spinae plane block versus serratus anterior plane block in pediatric patients undergoing aortic coarctectomy; a randomized controlled study
Source: BMC Anesthesiol. 2025 Jul 30;25:370. doi: 10.1186/s12871-025-03256-y (PMC12309128; doi:10.1186/s12871-025-03256-y)
Supplement: Supplementary file 4 — Supplementary Material 4. [file 12871_2025_3256_MOESM4_ESM.pdf]

CERTIFICATE OF ENGLISH EDITING

THIS DOCUMENT CONFIRMS THAT THE MANUSCRIPT LISTED BELOW WAS EDITED BY AN ENGLISH LANGUAGE EDITOR WHO HOLDS A MASTER'S DEGREE IN APPLIED LINGUISTICS AND WORKS AS AN ASSISTANT LECTURER IN THE DEPARTMENT OF APPLIED LINGUISTICS AND INSTRUCTION (TEFL), AIN SHAMS UNIVERSITY, AND PH. D CANDIDATE AT UNIVERSITY OF ALBERTA, CANADA.

I HEREBY CONFIRM THAT ALL LINGUISTIC PROBLEMS HAVE BEEN ADDRESSED, AS WELL AS THE FOLLOWING ISSUES HAVE BEEN CORRECTED: GRAMMAR, PUNCTUATION, SPELLING, SYNTAX, PHRASING, STYLE AND SENTENCE STRUCTURE.

Manuscript Title

ANALGESIC EFFICACY OF ULTRASOUND GUIDED ERECTOR SPINAE PLANE BLOCK VERSUS SERRATUS ANTERIOR PLANE BLOCK IN PEDIATRIC PATIENTS UNDERGOING AORTIC COARCTECTOMY; A RANDOMIZED CONTROLLED STUDY

Author

AHMED ALI MOHAMED GADO
ASSOCIATE PROFESSOR OF ANESTHESIOLOGY, SURGICAL INTENSIVE CARE AND PAIN MANAGEMENT DEPARTMENT, FACULTY OF MEDICINE, CAIRO UNIVERSITY, CAIRO, EGYPT

SIGNATURE

Marwa Abolfotouh

FOR CONTACT

abolfoto@ualberta.ca

Marwa.m.abolfotouh@women.asu.edu.eg

PES

PROOFREADING AND EDITING SERVICES

MA 121102023
